# Effectiveness of a home-based pulmonary rehabilitation maintenance programme: the Rehab2Life study protocol

**DOI:** 10.1186/s12912-024-01999-6

**Published:** 2024-05-21

**Authors:** Liliana Silva, Tiago Maricoto, Ângela Mota, Lara Lemos, Mariana Santos, Hélder Cunha, Inês Azevedo, Joana Berger-Estilita, Patrício Costa, José Miguel Padilha

**Affiliations:** 1 Matosinhos Local Health Unit, Matosinhos, Portugal; 2 CINTESIS, Centre for Health Technology and Services Research, Faculty of Medicine, University of Porto, Porto, Portugal; 3 Beira Ria Family Heath Unit/ULS Aveiro, Ílhavo, Portugal; 4 CICS-UBI - Health Sciences Research Centre & UBIAir - Clinical & Experimental Lung Centre, University of Beira Interior, Covilhã, Portugal; 5 Institute of Anaesthesiology and Intensive Care, Salemspital, Hirslanden Medical Group, Bern, Switzerland; 6 Institute for Medical Education, University of Bern, Bern, Switzerland; 7 Life and Health Sciences Research Institute (ICVS), School of Medicine, University of Minho, Braga, Portugal; 8 ICVS’3, PT Government Associate Laboratory, Braga, Guimarães, Portugal; 9 Faculty of Psychology and Educational Sciences, University of Porto, Porto, Portugal; 10 Porto Nursing School, Porto, Portugal; 11 CINTESIS@RISE, Porto, Portugal

**Keywords:** Pulmonary rehabilitation, Maintenance, Rehabilitation nursing, COPD, Home-based, self-managment, Functional capacity

## Abstract

Pulmonary rehabilitation (PR) is the bedrock of non-pharmacological treatment for people with COPD. Nonetheless, it is well described in the literature that unless the patient changes his behaviour, the benefits of PR programmes will decline in six to twelve months after finishing the programme. Therefore, maintenance programmes can address the problem of PR programmes’ effect loss over time.

Community care units can provide multidisciplinary care in the current Portuguese primary health care context. These units have an interdisciplinary team that aims to develop competencies in COPD patients to self-manage the disease.

This study aims to test the effectiveness of a 12-month home-based PR programme (Rehab2Life) compared to usual care through a single-blind randomised controlled trial with two parallel groups. The Rehab2Life programme includes two distinct phases. The first is an 8-week PR programme delivered to both groups, and the second is a PR maintenance programme delivered to the intervention group after the initial eight weeks. The control group receive the usual care and regular appointments. The primary outcome is functional capacity, and secondary outcomes are dyspnea, Health-Related Quality of Life (HRQoL), number of exacerbations, symptoms burden, anxiety and depression symptoms, and physical activity.

We expect to observe that the home-based PR programme brings clinically relevant benefits to the participants at the end of the first eight weeks and that, at 12 months after the maintenance phase of the programme, benefits are less dissipated than in the control group. We expect to identify the characteristics of the patients who benefit the most from home-based programmes.

The trial was registered on 7 April 2022 at ClinicalTrials.gov (NCT05315505).

## Introduction

Pulmonary rehabilitation (PR) is the cornerstone of non-pharmacological interventions for individuals dealing with the challenges of living with chronic obstructive pulmonary disease (COPD). PR’s key elements are exercise training, patient-directed education, smoking cessation support, disease self-management, and behaviour change, which are recommended as mandatory in standard COPD PR programmes. Moreover, PR has shown the ability to improve dyspnea and enhance exercise tolerance while evaluating and dealing with specific modifiable traits that can positively affect health-related quality of life (HRQoL) [1–4]. However, there is generally low access to PR programmes due to a lack of resources or referrals to these programmes [1–3]. Despite geographical disparities in the United States of America, overall, less than 4% of COPD patients have access to PR; in Canada, this is less than 1% [4]. Reporting to Europe, the problem remains [3], and in Portugal, only 2% of COPD patients have access to PR programmes. This scarcity might be attributed to impediments such as the absence of convenient access to specialised centres and geographical discrepancies [6, 8].

Exercise training and patient education in PR have been widely studied and are recommended by several guidelines and scientific societies [5–10]. However, self-management education and behavioural transformation often do not receive the attention they deserve from healthcare practitioners. It is paramount that specific treatable attributes, such as enduring breathlessness, exacerbations, and behavioural and social risk factors, occupy a pivotal position within any comprehensive PR regimen [3, 4].

The cardinal aspiration in managing chronic diseases is empowering patients to navigate their symptoms, treatments, and lifestyle alterations efficiently. Paradoxically, the means to instigate this empowerment remains obscure. Perceived health status and self-efficacy are essential for successful participation in PR programmes. These factors are crucial for developing the skills and knowledge needed to self-manage the disease, and they can often be the most difficult to address [11–13].

The PR programme’s benefits tend to wane after six months due to patients’ failure to adopt necessary behaviour changes [14–16]. Developing self-efficacy perception and self-management skills of the disease are vital issues to change patients’ health-related behaviours [5, 14, 17]. . However, this strategy alone has proven ineffective for all patients. Incorporating behavioral change techniques, such as physical activity diaries, is paramount to enhance the effectiveness of health interventions. These diaries can provide personalised and daily goals for each patient, ensuring a more effective path to change [18]. Therefore, action is needed to ensure that, after finishing PR programmes, patients will maintain disease control behaviours throughout time, their functionality level, and their health-related quality of life [19].

In response to the challenge of diminishing PR effects over time, the concept of PR maintenance programmes emerged as a promising solution [20–23]. It is also suggested that these programmes should be offered in a home-based setting, particularly to severe patients, for twelve or more months and should be supervised [24].

In the current Portuguese primary health care context, community care units can provide multidisciplinary care to patients with COPD. These units have a multidisciplinary team, including nurses specialists in rehabilitation, whose competencies enable prescribing and delivering PR programmes, as certified by the Portuguese Order of Nurses [25]. These community care units are geographically well-distributed across the country [26].

Therefore, rehabilitation nurses have an essential role in developing structured and multidisciplinary PR programmes that may achieve the needs of patients, even the most severe patients whose functional limitation is more pronounced and those more complex due to multimorbidity [27, 28].

### Objectives

The study aims to test the effectiveness of a 12-month home-based PR programme (the Rehab2Life programme) in maintaining functional capacity, health-related quality of life, impact of the disease and physical activity after a PR programme.

### Methods and analysis

Our study is a randomised controlled trial with two parallel groups. The Rehab2Life programme includes two distinct phases. The first is an 8-week PR programme delivered to both groups, and the second is an additional PR maintenance programme given to the intervention group.

### Study setting

**Fig. 1:**
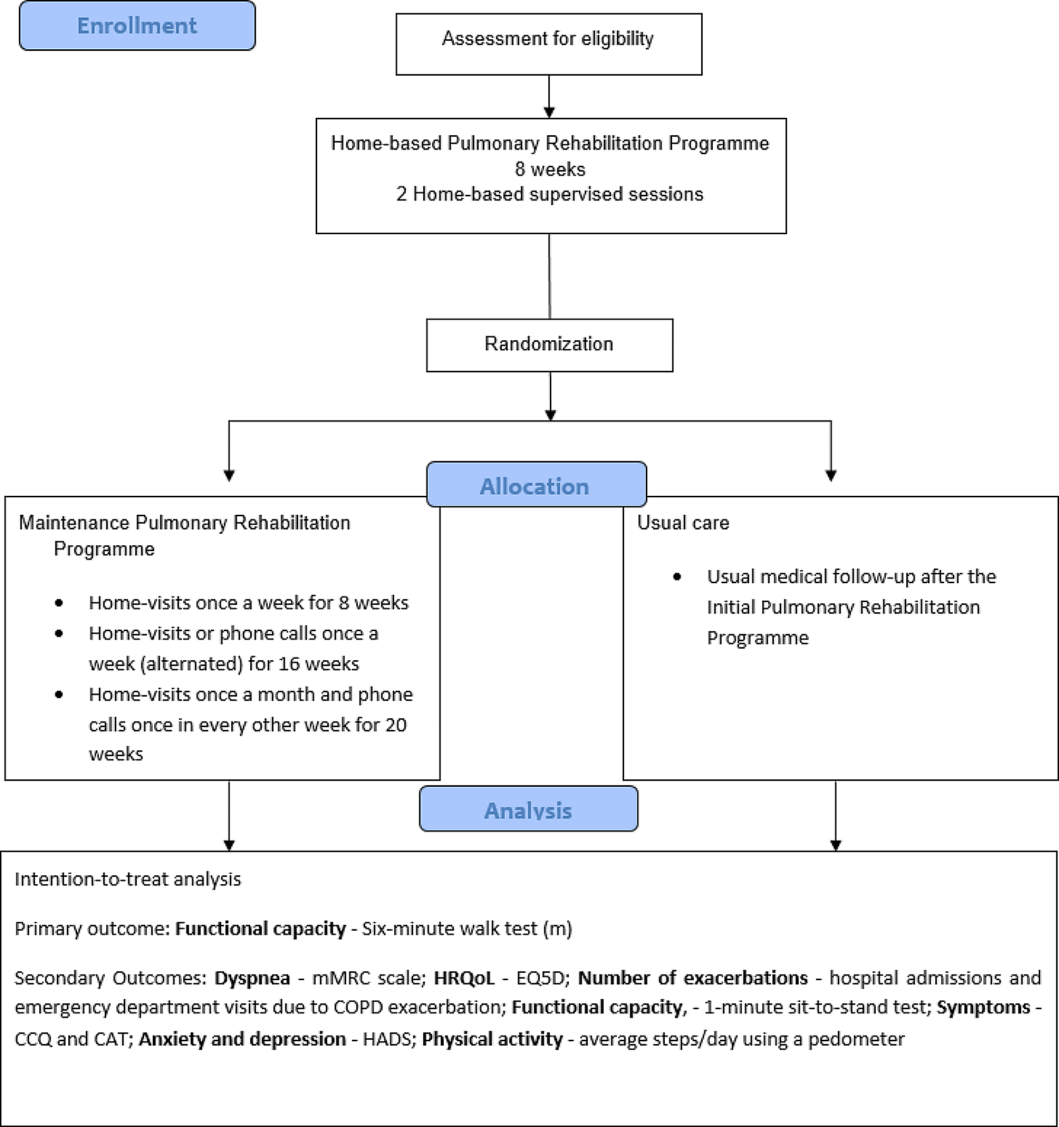
Study flow diagram

The study will occur in four community care units in a Portuguese urban area. All participants will undergo an 8-week home-based PR programme, after which one group will remain in a maintenance programme (intervention group). The other will have access to usual care (control group). Figure 1 – Flow diagram.

### Eligibility criteria

Patients classified as B or E, according to GOLD criteria (previously B, C and D) [23], residents in the area covered by the institution where the study is carried out will be eligible for inclusion.

We will exclude patients according to Portuguese General Directorate for Health guidelines, namely: History of a PR programme in the previous six months; Patients with a COPD exacerbation within the last week; Presence of unstable comorbidities such as comorbidities that limit exercise training: ischemic cardiopathy, unstable angina, severe aortic stenosis, hypertrophic cardiomyopathy, uncontrolled arrhythmia, decompensated congestive heart failure, uncontrolled diabetes, severe cognitive dysfunction or severe psychiatric disease interfering memory and adherence [29]; Score of the Clinical Frailty Scale 2.0 above six or above five in case of not having a responsible caregiver and living alone [30, 31] and oxygen saturation level (SpO_2_) below 85% in the 6-minute walk test [29].

We will include COPD patients referred by physicians to PR. After the referral and before the inclusion in the study, patients will be evaluated by a multidisciplinary rehabilitation team to assess clinical and safety conditions to perform home-based exercise training [29].

### Recruitment

The study will be presented to health professionals in primary care, pulmonology and internal medicine departments to promote referral to PR. A short training session will be given to remind health professionals about the benefits of PR and the importance of early and adequate referral.

### Assignment of interventions

After the patient’s informed consent, allocation concealment will be ensured by opening a well-sealed envelope. The trial was registered on March 21st 2022 at ClinicalTrials.gov (NCT05315505), and this protocol is reported according to the SPIRIT guidelines.

Outcomes will be measured at four time points (T0, T1, T2 and T3) with a cardiopulmonary technician, who will not be aware of the group allocation. Every assessment will take place in the regional hospital.

The first assessment (T0) will occur before the initial PR programme. After eight weeks, the subjects will be reassessed at T1 during randomisation. At seven months, corresponding to the intermediate period of the maintenance PR programme, the subjects will be reassessed at T2 and T3 at 12 months, corresponding to the end of the study.

### Intervention

Rehabilitation nurses are specialised nurses with background education in PR. Moreover specific training on the programme’s contents was given to them with the aim of uniformising protocol implementation.

The initial home-based PR programme will have two home visits every week. Every home visit will include a personalised educational intervention, supervised exercise training, self-management and self-efficacy strategies.

**Fig. 2:**
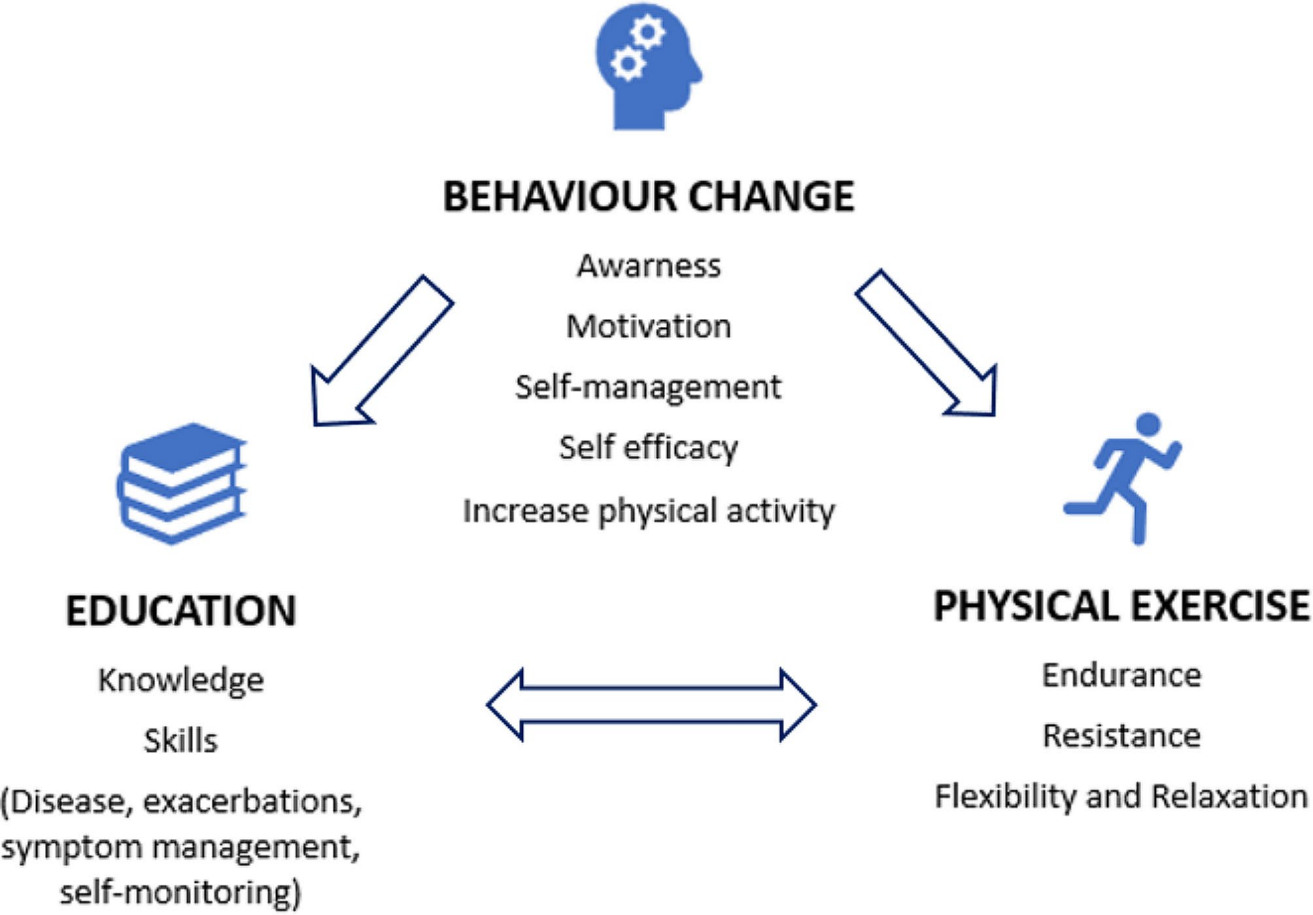
Main contents of the PR programme

The additional maintenance PR programme consists of home visits once a week for eight weeks, then home visits or phone calls once a week (alternated) for 16 weeks, and finally, home visits once a month and phone calls once every other week for 20 weeks (Fig. 2). Supervised exercise training will be conducted during home visits, validating the person’s knowledge and skills.

The intervention (Fig. 2) was designed after extensive literature review and external validation using focus groups. An exercise physiologist, a physical therapist, a specialist nurse, and a pulmonologist validated the physical exercise interventions. In addition, the educational content was validated by four specialist nurses who are widely experienced in PR programmes, and psychologists and highly experienced specialist nurses validated the self-management and self-efficacy strategies.

### Preparation

Before starting the Rehab2Life programme, patients go through a preparation phase in which they are taught functional respiratory re-education (FRR) techniques and issues related to awareness and motivation to perform physical exercise and changes in lifestyle habits are addressed.

FRR is essential when preparing for a PR programme in COPD patients due to limited exercise tolerance regarding their dyspnea and fatigue. FRR includes:

- Teaching patients pacing and breathing techniques that can help improve exercise tolerance [32];
- Diaphragmatic breathing, a type of breathing that can help to improve respiratory muscle function and reduce dyspnea [33].
- Pursed-lips breathing (PLB), that increases functional exercise tolerance, and it is a breathing technique that involves slowly exhaling through pursed lips. It is commonly used as a management strategy for COPD [34].

Traditional patient education programmes often assume that simply providing information will lead to behaviour change. However, this program recognises a critical limitation that education alone is insufficient [35] and does not improve exercise performance [14]. This program explicitly addresses this gap and prioritises fostering awareness and willingness to change alongside knowledge acquisition. Nonetheless, patient education is a crucial component of PR programmes, as it empowers patients with the knowledge and skills to manage their condition effectively and improve their HRQoL [35, 36]. .

Education.

The key elements of the educational component are synthesised in Table 1, where we organise the components of an education programme by category and dimension [37].

**Table 1:** Educational components of the Rehab2Life programme

Dimension	Category	Component
Self-management of COPD	Disease	COPD information
		Symptoms
		Evolution
		Importance of adapting lifestyle
		Limitations
		Travelling with COPD
		Sleep
		Sexuality
	Exacerbation	Symptoms identification
	Signs and symptoms management	Inhaled technique
		Oxygen therapy
		Dyspnoea
		Exercise intolerance
		Physical activity
		Anxiety
	Therapeutic regimen management	Smoking cessation
		Improve physical activity
		Medication
		Nutrition

This programme aims to give the patient a comprehensive understanding of COPD’s causes, symptoms, progression, and impact on daily life. Educating patients on the importance of a healthy diet and adequate hydration to support lung function and overall health is crucial to enhancing behaviour change [4, 38]. Implement evidence-based smoking cessation programmes to help patients quit smoking, as smoking cessation is essential for improving lung health and reducing the risk of complications, guiding gradually increasing physical activity and exercise, including specific exercises and modifications to accommodate COPD limitations, and equip patients with self-management skills to promote independent care and quality of life, addressing the emotional and psychological impact of COPD [4, 38].

Various educational methods include written materials, images and verbal instructions [39]. The Portuguese version of the Living Well with COPD™ is utilised [40]. The chapters are chosen according to the individual patient’s needs.

Behaviour change.

Encouraging active participation, self-reflection, and goal setting is fundamental to empowering patients and fostering motivation for behaviour change. In the Rehab2Life programme, the participants were encouraged to use goal setting to perform physical activity using an accelerometer (Yamax EX 510®) [41, 42].

In order to start the physical exercise component, some symptoms are ensured to be evaluated [5, 43, 44].

Patient assessment and monitoring:

- Monitoring at the Start and End of training: Blood pressure / HR / RR / Breathing pattern;
- Maximum HR during training calculation: 0.80*maximum HR achieved in the 6-minute walk test;
- For patients on beta blockers: Maximum Borg scale 4–5 in these patients associated with 50% of the HRR. HRR: Estimated maximum HR – basal HR. (Estimated maximum HR: 220-age);
- BP > 160/90 mm/Hg; SpO2 < 90%; RR > 22respiratory count/min the patient does not start training;
- Continuous monitoring - SpO2 and HR – If LTOT, adjust to prescribed flow on exertion;
- If SpO2 at rest < 90% the patient does not start training.
- Assess the presence of dizziness, headache, fever, chest pain, and musculoskeletal pain (except mild to moderate post-workout muscle pain) – If present, the patient does not continue the training.

Physical exercise.

**Table 2:** Exercise training components of the Rehab2Life programme

Exercise training components	
Warm Up	- Bilateral neck flexion, shoulder rotation, elbow flexion, wrist flexion/extension; - Hip and knee flexion (walking)
Endurance training	- Walking - Cycling - Stairs
Muscle strengthening	- Hip flexion/abduction and knee flexion in standing position - Seated knee extension - Squats - Full elbow flexion - Shoulder press - Push-ups on the wall
Cool-down	- Stretching the muscle groups recruited in the session

The FITT principle, which stands for Frequency, Intensity, Time, and Type, provides a framework for prescribing exercise training that effectively overloads the body. Exercise intensity for people with COPD is typically based on symptoms, and a commonly used tool is the modified Borg scale, which ranges from 0 (no exertion) to 10 (maximal exertion). A maximum exertion level of 6 on the Borg scale during exercise is often recommended in a home-based setting. Gradually increasing the duration of sessions over time is an effective way to overload the body and promote physiological adaptations. The type of exercise (Table 2) should be chosen based on the individual’s interests, preferences, availability, and safety considerations [9].

The patients must understand and monitor safety symptoms as they are motivated to perform physical exercise at least one day a week with no supervision.

The research team’s experience with COPD patients at home revealed that after finishing a centre-based program, patients often stop exercising with equipment (as most lack it at home). This leaves them with only walking and breathing exercises. To address this, we aim to give patients home tools and alternatives so they can continue the PR programme with their own resources.

Patients will be referred to a nutritionist if the Malnutrition Universal Screening Tool (MUST) score exceeds two or the Strength, Assistance in walking, Rise from a chair, Climbing stairs, and Falls score (SARC-F) exceeds four [45, 46]. The referral to a social worker will be done if the patient shows economic issues that compromise access to controlled medication, and patients will be referred to a medical doctor if there are any new exacerbation symptoms.

The programme will be interrupted in a COPD exacerbation until the condition is resolved. After being clinically stable and exhibiting minimal symptoms when compared to the usual state, the participant will receive six more supervised home PR sessions and then resume the protocol in the phase in which it was interrupted [47].

The usual care group will receive their usual medical follow-up after the initial PR programme.

All the rehabilitation nurses delivering the Rehab2Life programme have had specific training on all components of the programme, records and monitoring that must be carried out, ensuring patient safety.

### Outcomes

#### Primary outcome

The primary outcome in this study is the individual’s functional capacity, assessed by the distance in meters the patient can walk over 6 min, performing the 6-minute walk test. The 6mWT is a predictor of morbidity and mortality among COPD patients, it is a simple clinical exercise test for the objective evaluation of functional exercise capacity improvement in COPD, which measures the distance a patient can quickly walk on a flat hard surface in 6 min [48, 49]. .

### Secondary outcomes

The secondary outcomes are dyspnea, Health-Related Quality of Life (HRQoL), exacerbations, symptoms, anxiety and depression, and physical activity.

Dyspnea will be assessed by the modified Medical Research Council (mMRC) scale [32]. This is a widely used scale with four items in which the higher the value, the greater the dyspnea intensity.

HRQoL will be assessed by the Eq. 5D [50]; The EuroQol instrument is intended to complement other quality-of-life measures and to facilitate the collection of a common data set for reference purposes.

The number of exacerbations measured by hospital admissions and emergency department visits due to COPD exacerbation will be evaluated via consultation of electronic records [51];

Symptoms will be measured by the Clinical COPD Questionnaire (CCQ) [52, 53] and the COPD Assessment Test (CAT) [54];

Anxiety and depression will be assessed by the Hospital Anxiety and Depression Scale (HADS) [55];

A pedometer (Yamax EX 510®), validated in COPD, will assess physical activity by monitoring the average daily steps [17, 41, 56]. Participants will wear the device for seven consecutive days during waking hours (except when bathing or sleeping).

Regular physical activity (p.a.) is associated with important health benefits and may prevent the development and progression of chronic diseases.

### Other measures

We will quantify the number of times an intervention is carried out and how long it takes to achieve the intended effect.

### Sample size and statistical methods

The minimum sample size required was calculated considering the final comparison between the two groups regarding the primary outcome and the inclusion of five covariates to adjust for potential comparison biases between the groups.

We will perform an intention-to-treat analysis. Therefore, stipulating ANCOVA as the statistical procedure to be used, a significance level of 5%, a test power (1-β) of 80%, an average effect magnitude (f = 0.25, corresponding to $\eta$^2^= 0.06, 6% of the variability of the dependent measure under evaluation to be explained by the group) and five covariates in the model, the sample size to be collected will be 128 participants, which corresponds to 69 participants for each group.

Considering that we may have 20% follow-up losses, the sample size to be collected will be 160 participants (80 per group).

### Data analysis

The sample size covers the sample dimension necessary for applying a mixed ANCOVA. The group will be considered an inter-subject factor, the time/intervention (four moments) as the intra-subject factor, and the five covariates. This analysis will test the main and interaction effects between these two factors.

We also aim to carry out the described procedure using only the three moments T1, T2, and T3 and consider T0 an additional covariate. We will proceed with their decomposition if significant time or interaction effects exist.

Finally, a latent growth model will be tested to assess the trajectory (linear and quadratic) of the study participants concerning outcomes and how this trajectory is conditioned by the factors that originate the groups.

Statistical analysis will be performed using IBM SPSS Statistics V28 and IBM SPSS Amos V28, and p values < 0.05 will be considered significant.

## Discussion

Real-world studies with PR programmes in low-resource settings have evaluated the evolution of functional capacity and QoL of home-based programmes without support equipment [19, 57]. Such studies concluded that a home-based PR programme, using minimal resources and little direct supervision, delivers improvements in functional exercise capacity and HRQoL that are at least equivalent to conventional centre-based PR in people with COPD [19, 57]. Not providing equipment to participants is a methodological option since the objective of this study is to evaluate effectiveness, which differs from efficacy. We want to evaluate an intervention in the real-world context, where people do not all have access to the necessary equipment and still have to continue living with their disease, developing their own strategies with the nurse’s guidance. It is essential to set realistic goals so that COPD patients start slowly and gradually increase the duration and intensity of their workouts over time. Self-monitoring is vital so that patients can identify the symptoms related to the risk of complications [4, 43].

According to the study design, we expect to observe that the home-based PR programme brings clinically relevant benefits to the participants at the end of the first eight weeks and that, at 12 months, after the maintenance phase of the programme, the benefits obtained after the eight weeks fade away slower than individuals who do not have the programme maintenance phase.

It is also expected that patients experience fewer COPD exacerbations.

### Main potential biases

The intervention is unlikely to be blinded, which can be considered biased.

Another aspect that could bias our study is the Hawthorne effect throughout the study (i.e. behaviour change in participants due to their involvement in the study). However, establishing a cohort time of one year will not sustain this effect. On the other hand, the control group (“usual care”) will maintain their usual care with their assistant doctors, who are entirely free from any influence of the study design. With this approach, the Hawthorne effect will not contaminate the control group and represent usual real-life care.

Moreover, we also expect some attrition bias, considering the length of the follow-up and the need for complete adherence to the study protocol, mainly the intervention. Nevertheless, by increasing the sample size to a potential loss to follow-up up to 20%, we expect to overcome it.

Another potential bias is related to our inclusion/exclusion criteria. Since we excluded patients with complex and critical comorbidities, we cannot extend our conclusions to the general COPD population. However, this ensures a proper and exact evaluation of the intervention’s potential benefit. That might, to some extent, apply to those patients as well.

We expect that this work will contribute towards the preservation of the benefits obtained after a classic PR programme (8-weeks), through implementation of a maintenance component. At the same time we expect to increase the availability of this essential treatment to COPD patients and to identify the characteristics of the patients who benefit the most from home-based programmes.

## Data Availability

No datasets were generated or analysed during the current study.

## Declarations

### Trial status

Recruitment has started and is ongoing.

## Abbreviations

PR: Pulmonary rehabilitation
COPD: Chronic obstructive pulmonary disease
HRQoL: Health-related quality of life
MUST: Malnutrition Universal Screening Tool
SARC-F: Strength, Assistance in walking, Rise from a chair, Climbing stairs, and Falls score
mMRC: modified Medical Research Council
CCQ: Clinical COPD Questionnaire
CAT: COPD Assessment Test
HADS: Hospital Anxiety and Depression Scale
FRR: Functional respiratory re-education
PLB: Pursed-lips breathing
HR: Heart rate
HRR: Heart rate reserve
